# Fixed Bed Chemical Looping beyond Gas Switching: Application to Dynamic Industrial Waste Gas Conversion

**DOI:** 10.1021/acs.iecr.6c00301

**Published:** 2026-05-27

**Authors:** Andrew J. Furlong, Nicole K. Bond, Jan B. Haelssig, Christopher de Leeuwe, Adam Zaidi, Michael J. Pegg, Vincenzo Spallina, Robin W. Hughes

**Affiliations:** † Natural Resources Canada, 113679 CanmetENERGY-Ottawa, 1 Haanel Drive, Ottawa, ON K1A 1M1, Canada; ‡ Department of Process Engineering and Applied Science, 3688 Dalhousie University, P.O. Box 15000, Halifax, NS B3H 4R2, Canada; § Department of Chemical Engineering, 5292 University of Manchester, Manchester M13 9PL, United Kingdom; ∥ Department of Chemical and Biological Engineering, 120472 University of Ottawa, Ottawa, ON K1N 6N5, Canada

## Abstract

This article presents first-of-their-kind investigations on fixed bed chemical looping as a means of converting dynamic industrial process waste gases containing CO, H_2_, or hydrocarbons to a product suitable for delivery to a CO_2_ separation system, without dilution by excess combustion air or flaring outside of process lines. The electric arc furnace (EAF) is used as a case study for its extreme variability in composition and flow rates, and a sample process configuration is presented. Copper- and iron-based oxygen carrier (OC) materials are tested using batched synthetic CO-rich EAF off-gas at the bench scale in a fixed bed reactor to assess the operating limits of the materials with respect to temperature, flow rate, and OC conversion. Results show high OC utilization is possible before breakthrough, with potential for increases in higher-temperature reactors. External mass transfer resistances and possible flow channeling were observed in the bench-scale system and their elimination in pilot and full-scale reactors is discussed. Coke deposition occurred on the iron-based material and resulted in minor deactivation; however, pathways for elimination via temperature management and the use of a small supply of steam with feed gases are described. A selection of processes with dynamic product or waste streams that may benefit from fixed bed chemical looping as a waste treatment or emissions reduction system are introduced, highlighting the absence of treatment methods in use. A fixed bed chemical looping reactor configuration utilizing external heat removal is proposed for load balancing.

## Graphical abstract

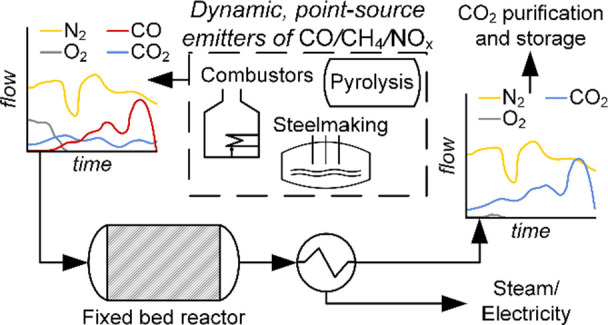

## 1 Introduction

Chemical looping, typically implemented as chemical looping combustion (CLC), is a novel energy technology, using a metal/metal oxide pair known as an oxygen carrier (OC) to move oxygen between air and fuel reactors, most commonly in interconnected fluidized bed arrangements.^1^ This technology has the benefit of reducing the energy requirements for separation of CO_2_ from N_2_ present under air-fired combustion, while still using traditional fuels, including natural gas and coal.^2^ Alternatively, biomass can be used as a fuel and can make net-negative operations possible when coupled with carbon capture, utilization, and storage (CCUS).^3^ Chemical looping offers an alternative to traditional fired combustion, especially in applications where electrification is not feasible. These reactors operate under extreme conditions, with pilot-scale reactor temperatures of up to 1050 °C reported in the literature.^4^ Alternative designs for chemical looping have been investigated, including fixed bed reactors utilizing gas switching,^5^ and rotating reactors.^6^

Chemical looping in fixed bed reactors was originally proposed as a means of removing the fluid-particle separation which is inherent to fluidized bed CLC.^5^ Subsequent investigations have demonstrated fixed bed CLC is viable with gaseous fuels, alternating between two gases.^7,8^ However, the focus of experimental work at the bench scale has been the production of syngas or H_2_, utilizing steam or CO_2_ as an oxidizer, or exploiting the catalytic behavior of some OCs, in a three-stage process.^9–11^ These processes are inherently dynamic, but their feed gases use two or three fixed flow, fixed composition inlet conditions; continually varying flow rates and compositions have not been investigated. Of particular relevance to this work are the recent investigations utilizing blast furnace off-gases to concurrently produce H_2_ and a moderately concentrated CO_2_ product (potentially exceeding 50%), as a part of the C^4^U project.^12,13^ Present challenges preventing the scale up of fixed bed chemical looping include limitations in fast, high-temperature gas switching, and the need for multiple parallel reactors for stability in downstream systems.^14^

The electric arc furnace (EAF) is a primary steelmaking pathway, representing approximately one-third of global steel production,^15^ and allows for green energy consumption to melt and refine steel.^16^ Although the emissions associated with heating can be cut by this process when compared to the blast furnace-basic oxygen furnace route, there are still direct emissions from the use of natural gas burners and/or coke, accounting for an estimated range of 37 kg CO_2_e (t steel)^−1^ to 1080 kg CO_2_e (t steel)^−1^.^17–19^ Current best practices globally have equivalent emissions of 330 kg CO_2_e (t steel)^−1^ from EAF routes, when coupled with green energy,^20^ indicating that a renewables-powered future can have very low overall emissions when coupled with capture of emissions from produced gases. Kirschen et al.^19,21–23^ have further examined the off-gas from EAFs, finding losses of up to 35% of the total energy input via the off-gas, with a sizable fraction from the incomplete combustion of CO and H_2_. The time course of these off-gases is cyclic, where excess air (and therefore O_2_) is present in the off-gas early in a cycle, and levels of CO up to 65% and H_2_ up to 20% are recorded in later stages of the cycle, which typically varies between 30 and 80 min, depending on the facility. When CO or H_2_ are present, free O_2_ is at a minimum, if detectable. The flow and composition profiles reported by Kirschen et al.^21–23^ closely align with the feed used in fixed bed CLC with gas switching, however with a more gradual change between oxidizing and reducing feed, and heat removal replaced by extended oxidation. Presently, recovery of this waste heat and capture of these direct emissions are not practiced to any meaningful level, owing to these compositional changes, highly variable flow rates, and the extreme temperatures of the produced gases.

Fixed bed chemical looping is an inherently dynamic process that may be well suited to treat a complex waste gas stream having a dynamic composition and flow rate. Therefore, this work proposes a process and presents the first investigations on chemical looping combustion as a novel method to treat EAF off-gas and capture CO_2_ from industrial processes. The modeled EAF off-gas stream is divided into seven stages—rather than the conventional two or three—representative of the dynamic behavior of an industrial system. The response of a copper-based material and an iron-based material are shown, demonstrating the effects of operating temperature and flow rate on the breakthrough of CO. The stability of the two materials is discussed, especially regarding coke formation and implications on process scaling. Finally, the broader application of fixed bed chemical looping as a means of processing other industrial waste gases that have dynamic variability, such as those from raw materials and power production, is introduced. The benefits of using chemical looping to convert carbon-containing species to CO_2_ for integration with CCUS, and also of eliminating residual CH_4_ or NO_*x*_ for atmospheric release from existing combustors without the use of precious metal catalysts are discussed.

## 2 Methodology

### 2.1 Apparatus

The experimental apparatus is shown in Figure 1 and generally described by Figure 2. This system is intended for small quantities of OC (approximately 1–10 g) and low gas flow. The system is contained within a furnace. Heavy insulation is installed around the furnace and reactor. The reaction vessel is constructed of 253MA, with an inner diameter of 15.8 mm (1/2-in. sch. 40 pipe). The OC is supported atop a small piece of quartz wool and is held approximately centered in the vessel by a bed of inert Al_2_O_3_; no temperature, flow, or compositional measurement instruments are in the bed. Flow rates are controlled by mass flow controllers (Bronkhorst EL-FLOW Select, 200 standard cubic centimeter per minute [SCCM] capacity referenced to 20 °C and 101.3 kPa). Gases are supplied to the top of the reactor and are preheated in the void space in the reactor above the OC.

**Figure 1:**
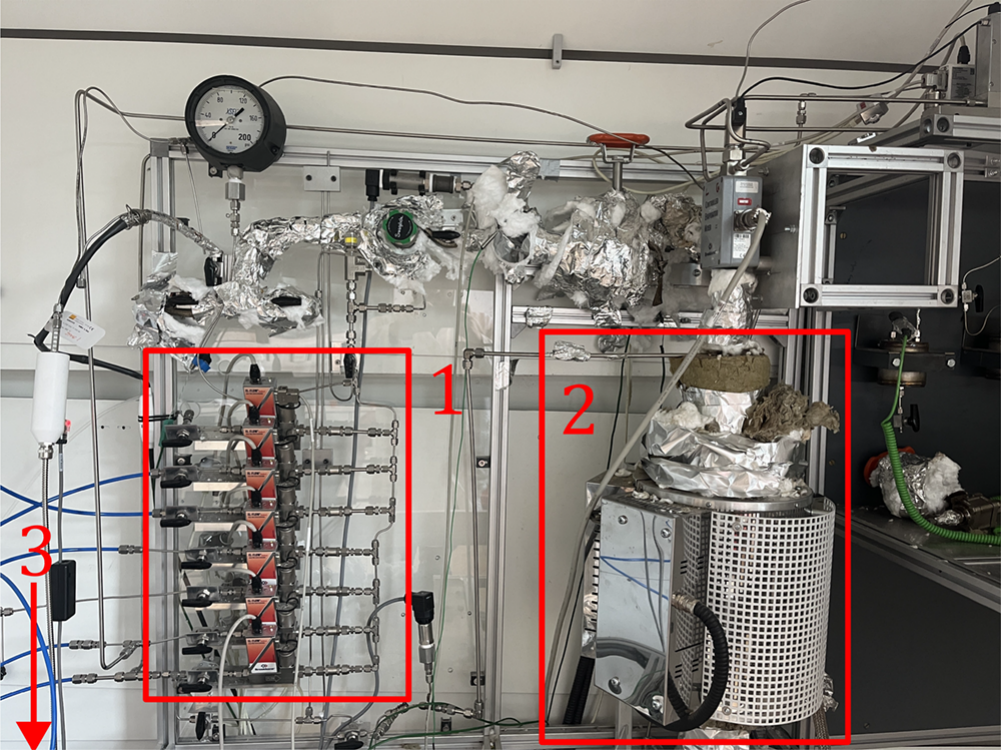
Experimental apparatus as tested in this work. Flow control is provided by the mass flow controllers (left), and the reactor is contained within a vertical furnace (right). (1) Flow control; (2) reactor; (3) mass spectrometer analysis.

**Figure 2:**
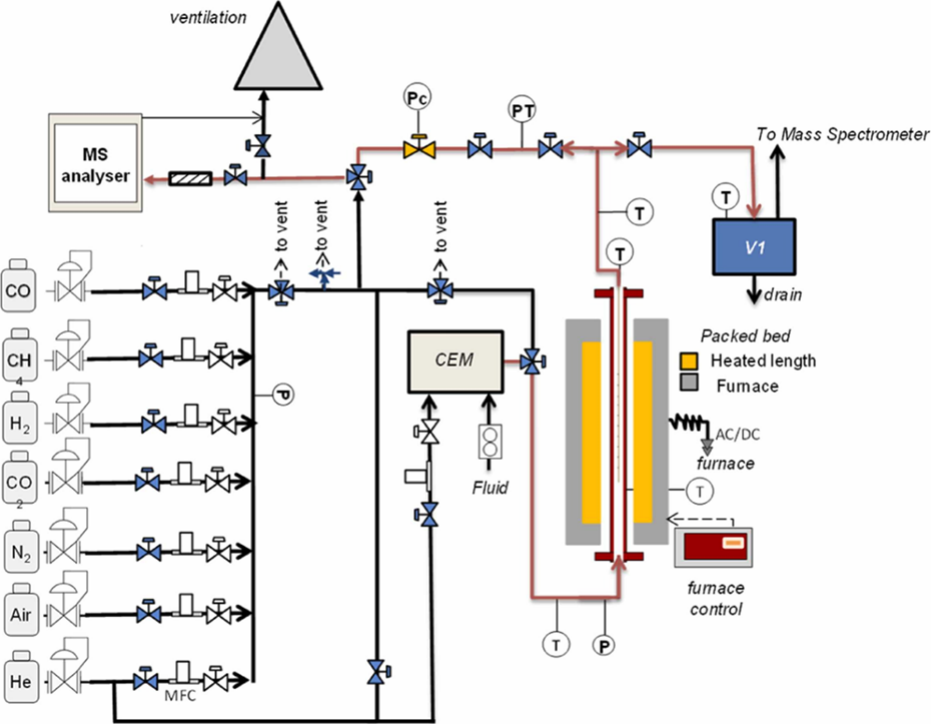
Schematic diagram of the experimental apparatus used in this work. MS: mass spectrometer; CEM: controlled evaporation mixing (not used). Reproduced from Zaidi et al.^24^ Available under a CC BY-NC-ND license. Copyright 2024 Adam Zaidi, Christopher de Leeuwe, Sarayute Chansai, Christopher Hardacre, Arthur Garforth, Christopher Parlett, and Vincenzo Spallina.

### 2.2 Materials

#### 2.2.1 Oxygen Carrier

Two OCs were examined in the bench scale apparatus. Structural and chemical details on these two materials cannot be disclosed due to confidentiality agreements.

The first material investigated primarily contains CuO and has previously been tested in other work.^25,26^ This material was sieved to a size range of 710 to 1200 μm and 5.00 g was loaded into the reactor in its oxidized form, creating a bed with a length of approximately 32 mm (*L*/*D* ≈ 2.0). This material is shown in Figure 3a. Upon first heating, it was noted that a small portion of the OC mass was adsorbed water, indicated by a brief 18 Da signal in mass spectrometer (MS) analysis of the gas flow out of the reactor.

**Figure 3:**
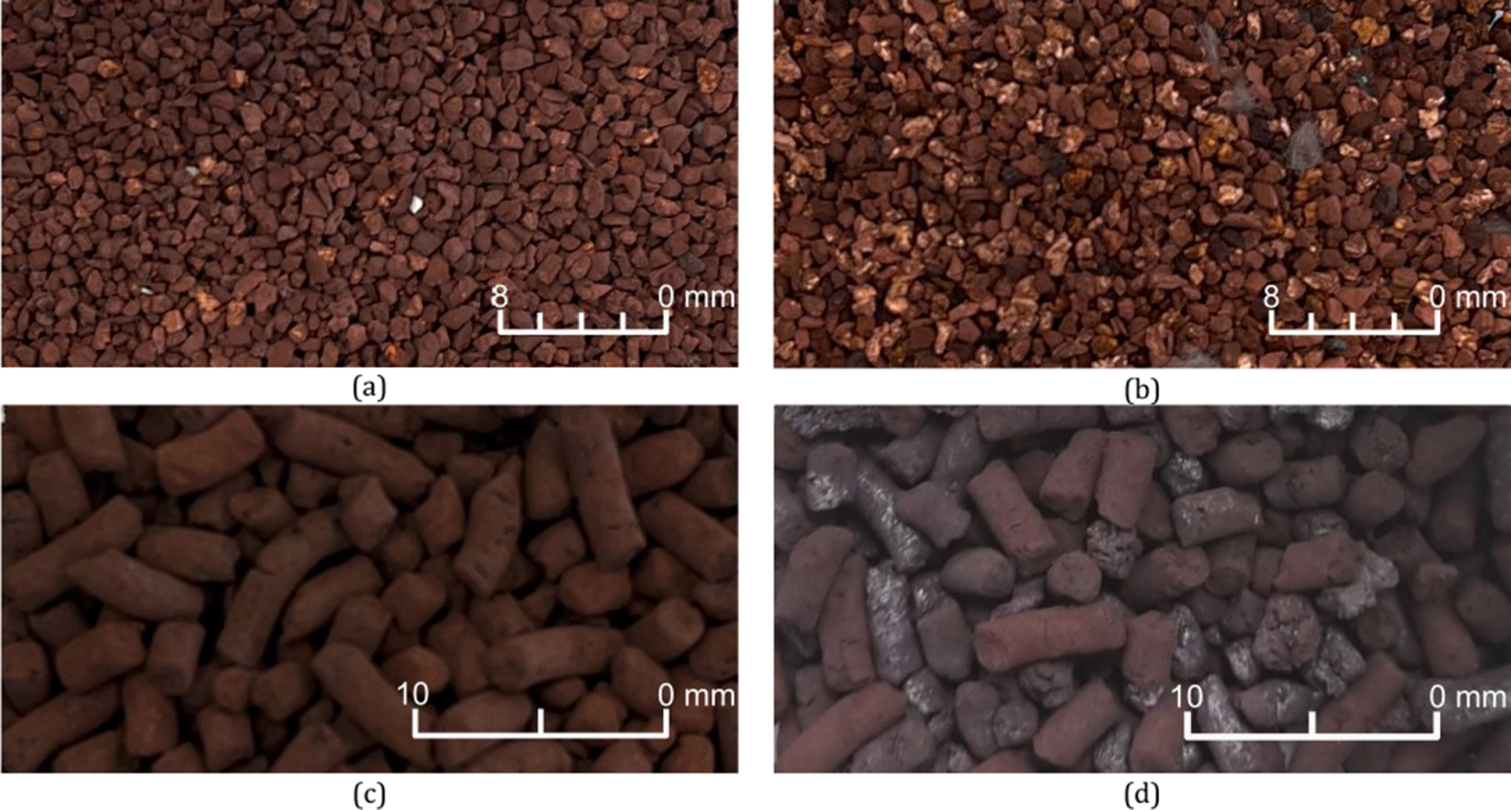
Unused and used oxygen carrier materials tested in this work. (a) Unused copper-based material, (b) used copper material, (c) unused iron material, and (d) used iron material.

The second material examined was a waste-derived product, primarily containing scrap from a steelmaking facility. The oxygen transport capacity of this material has been measured by thermogravimetric analysis (TGA) to be 8.0%. This aligns with composition measures (method not reported by the scrap supplier) of approximately 80% of the raw material being Fe_2_O_3_ and the remainder a mixture of metal oxides and SiO_2_; near-total availability of the oxygen contained within Fe_2_O_3_ on a transition to FeO is indicated. This material was prepared as a cylindrical extrudate with a diameter of 2 mm, calcined at 950 °C under air, and crushed to form a mixture of short and long cylinders. The material was sieved to remove fine dust generated by the crushing process using a 710 μm sieve, and 10.01 g was loaded in the reactor in its oxidized form, with a bed length of approximately 50 mm (*L*/*D* ≈ 3.2). This material is shown in Figure 3c.

#### 2.2.2 Gases

Gases were supplied to the reactor from building supplies (air, N_2_, He), and from cylinders (CO—99.5% compressed gas [BOC], CO_2_—99.5% liquified gas [BOC]). Building gas supplies were CP grade (99.5%) or higher purity.

### 2.3 Analytical

Product gas compositions were analyzed by MS (Hiden Analytical HPR-20) at a frequency of 2 Hz. Molecular masses included in analysis were 2 Da (H_2_), 4 Da (He), 15 Da (CH_4_ → CH_3_ fragmentation), 18 Da (H_2_O), 28 Da (N_2_, CO, and some CO_2_), 32 Da (O_2_), and 44 Da (CO_2_). CO was detectible above 15 ppm. The delay time between feed changes and detection by the MS varied from 20 to 40 s, depending on the flow rate into the reactor, operating temperatures, composition of the feed, and conversion of the reactor bed.

At the start of each experimental day, calibration with a minimum of 210 s per species was used. Calibration gas mixtures were supplied through the reactor at ambient temperatures, by the flow control system. Levels used were 10.5% O_2_ (diluted air, with 50% He), 50% CO (balance He), and 75% CO_2_ (balance He). A fraction of the 28 Da signal known to originate from fragmentation of CO_2_ was accounted for using lab standard procedures. H_2_, CH_4_ and H_2_O levels were not regularly calibrated because there was no supply of H to the reactor in any species.

Because both N_2_ and CO appear as 28 Da signals, it was assumed that the supplies of air, He, and CO_2_ were free of CO, and the supplies of He, CO and CO_2_ were free of N_2_. This allowed identification of the 28 Da signal as N_2_ or CO, depending on the feed gas. Flow rates for each species were calculated using the feed flow rate of He, and the measured concentration of He and each species of interest in the product gas.

### 2.4 Operations

The reactor feed, which represents an EAF off-gas stream, is based on the work of Kirschen et al.^21^ and is given in Table 1. One complete cycle of the reactor consists of seven stages. The first two stages represent charging the furnace and initial melting, with gases being approximately comprised of air and rising CO_2_ levels. The third and fourth stages show the variable levels of CO and CO_2_ during the oxygen blowing and refining stages, as coke is consumed by pure O_2_ supplied to the furnace. Stage 5 represents pretapping, when the furnace canopy is open and the EAF exhaust is drawing in ambient air. Finally, stages 6 and 7 represent final refining, when the furnace canopy is again closed and CO flow rates spike, before tapping (removal of molten steel).

**Table 1:** Simplified EAF Off-Gas Stream Based on Analysis of the Dynamic Composition and Flow Profiles Reported by Kirschen et al.^21^

		total volumetric flow rate (SCCM)										
		copper			iron				volume fraction (%)			
stage	*t*/*t*_ref_ ^a^	low	medium	high	low	medium	high	high–high	air	He	CO	CO_2_
1	0.24	200.8	251.0	251.0^b^	167.0	191.0	251.0	251.0^b^	79.7	12.0	0.0	8.4
2	0.20	256.8	321.0	321.0^b^	215.0	245.0	321.0	321.0^b^	43.3	46.1	0.0	10.6
3	0.20	220.0	275.0	314.3	183.0	210.0	275.0	314.3	0.0	61.8	28.3	9.8
4	0.16	117.6	147.0	168.0	98.0	112.0	147.0	168.0	0.0	77.6	14.3	8.2
5	0.06	208.8	261.0	298.3	174.0	199.0	261.0	298.3	66.7	19.5	0.0	13.8
6	0.10	260.8	326.0	372.6	218.0	239.0	326.0	372.6	0.0	56.5	33.3	10.2
7	0.04	103.2	129.0	147.4	86.0	99.0	129.0	147.4	0.0	81.4	10.9	7.7

a *t*_ref_ = 50 min for copper, 25 min for iron, and 28.6 min for iron with extended cycles.

b No change in flow rate because flow controller capacity was reached; full oxidation was still achieved.

Multiple flow levels were used to test the range of expected velocities for full scale systems for their effects on breakthrough. Tests with the copper-based material were operated for a base time of 50 min, with a shortened oxidation window (stages 1 and 2) when compared to the referenced gas supply because of the excess air present in operations, and limits in lab use hours. A total of 36 cycles were conducted for the copper-based material. For the iron-based material, cycles were shortened to a base of 25 min to maintain comparable flow rates to the copper-based material, with a reduced quantity of oxygen available for reactions in the bed. The reduced reactivity of the iron-based material was accounted for by increasing reactor temperatures. Additional studies were conducted with a 14% increase in cycle times, to 28.6 min, for the high flow rate case (matching the totalized gas flow of the high–high flow case), to evaluate if bed conversion or advective dispersion was the dominant source of breakthrough for the tested conditions. A total of 45 cycles were conducted for the iron-based material.

Flow rate changes were manually completed, requiring approximately 15 s to update all flow controllers. The order of all trials with their conditions and number of cycles is given in Table S1. The gas supply has been adapted considering the limits of the equipment and operating schedules. No H_2_ was included in the feed as the apparatus could not reliably supply the low flow rates which would be required for its trace presence (approximately 0.6 mol % of the total feed). The elimination of H_2_ from the reactor feed was not balanced by any additional inert or reducing feed because it represented such a small portion of the totalized reducing flow and total flow overall and would have its presence masked by the batching applied to the sample gas stream. When CO was supplied to the reactor, no air was supplied for safety and for differentiation of CO and N_2_. Additionally, the N_2_ present in the flue gas presented by Kirschen et al.^21^ during reducing windows was replaced by He to maintain the same concentration of CO and gas velocity without altering the 28 Da MS signal. Although He and N_2_ have different specific heat capacities and N_2_ is not entirely inert, with the low gas velocities, limited theoretical maximum temperature rise, and low temperatures for NO_*x*_ formation this change was assumed to have no effect on results.

### 2.5 Process Implementation

For an electric steelworks, chemical looping is proposed to be implemented in a configuration similar to Figure 4. Under this configuration, gases are pretreated, with liquid water cooling to remove dust and reduce gas temperatures from 1900 °C or higher, to more manageable temperatures (500–800 °C). This cooled gas stream is then passed through a chemical looping reactor for oxidation and reduction, depending on the current upstream feed, and is cooled by boiler feedwater to recover heat from the hot product gas in a usable stream. The recovery of this heat is an essential component of the process, especially in the EAF, where one of the most notable sources of energy inefficiencies is in the absence of heat recovery from off-gas.^27^ Depending on the reactor configuration, direct heat removal may also be possible or required to manage reactor temperatures; the use of a cooling loop and heat sink rather than direct removal to steam or another working fluid is recommended for process smoothing with a dynamic load. In other contexts, alternative pretreatment may be required, such as a scrubbing system for the removal of hazardous species (e.g., halocarbons), or the addition of heat to bring reactants to typical chemical looping reaction temperatures when handling low-temperature waste gases.

**Figure 4:**
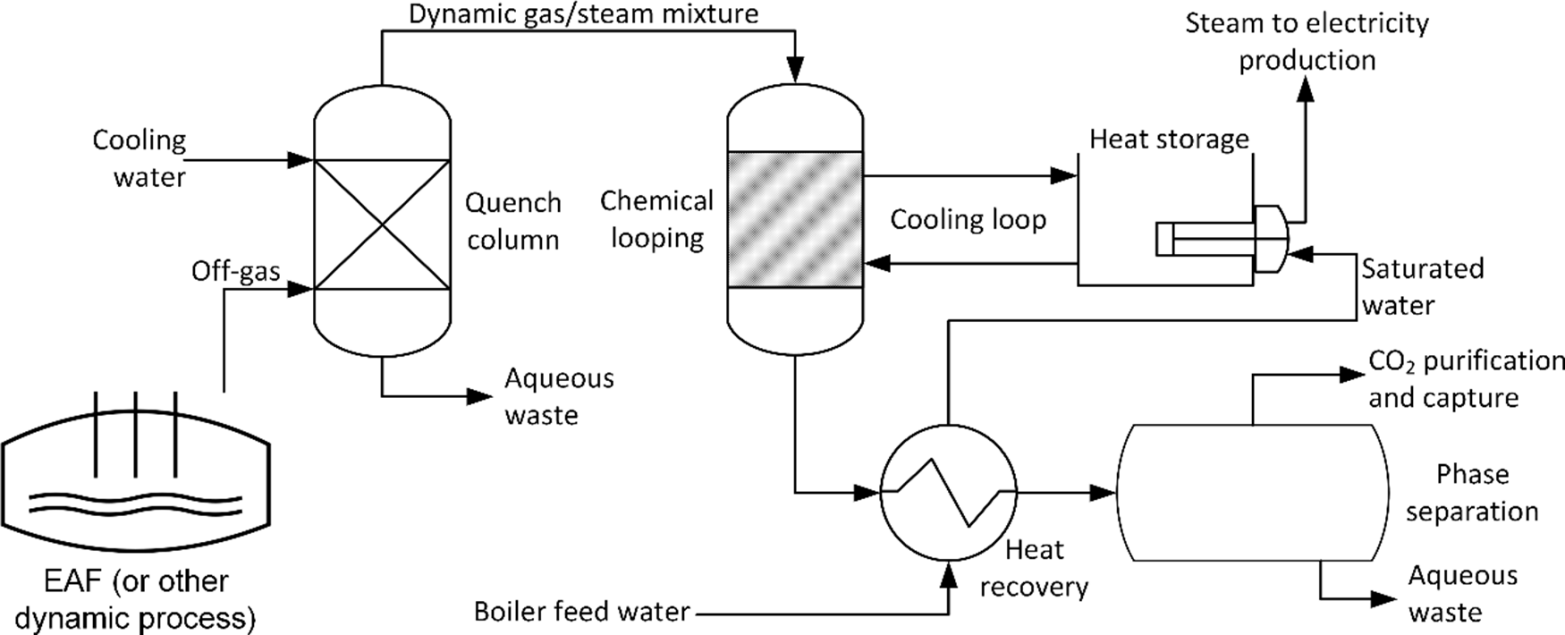
Proposed process configuration for implementing fixed bed chemical looping in a dynamic plant like an EAF facility.

## 3 Results

### 3.1 Gas Breakthrough

Product gas composition for a complete seven-stage cycle is shown in Figure 5. Figure 6 presents the level of CO present in the off-gas, reported as molar fractions, as a function of time throughout representative cycles for the copper-based material under different flow rate and temperature combinations. The corresponding decrease in CO_2_ exiting the reactor is seen in Figure 7 for the high flow rate cases. Figure 8 shows the effects of temperature on CO breakthrough using the iron-based material, under the high–high flow condition. Figure 9 shows the breakthrough of CO for different flow rates, and an extended cycle timing, when operating at 700 °C. These results do not include the first 40 s of each cycle due to the sensor delay from the void volume in the reactor and supporting piping.

**Figure 5:**
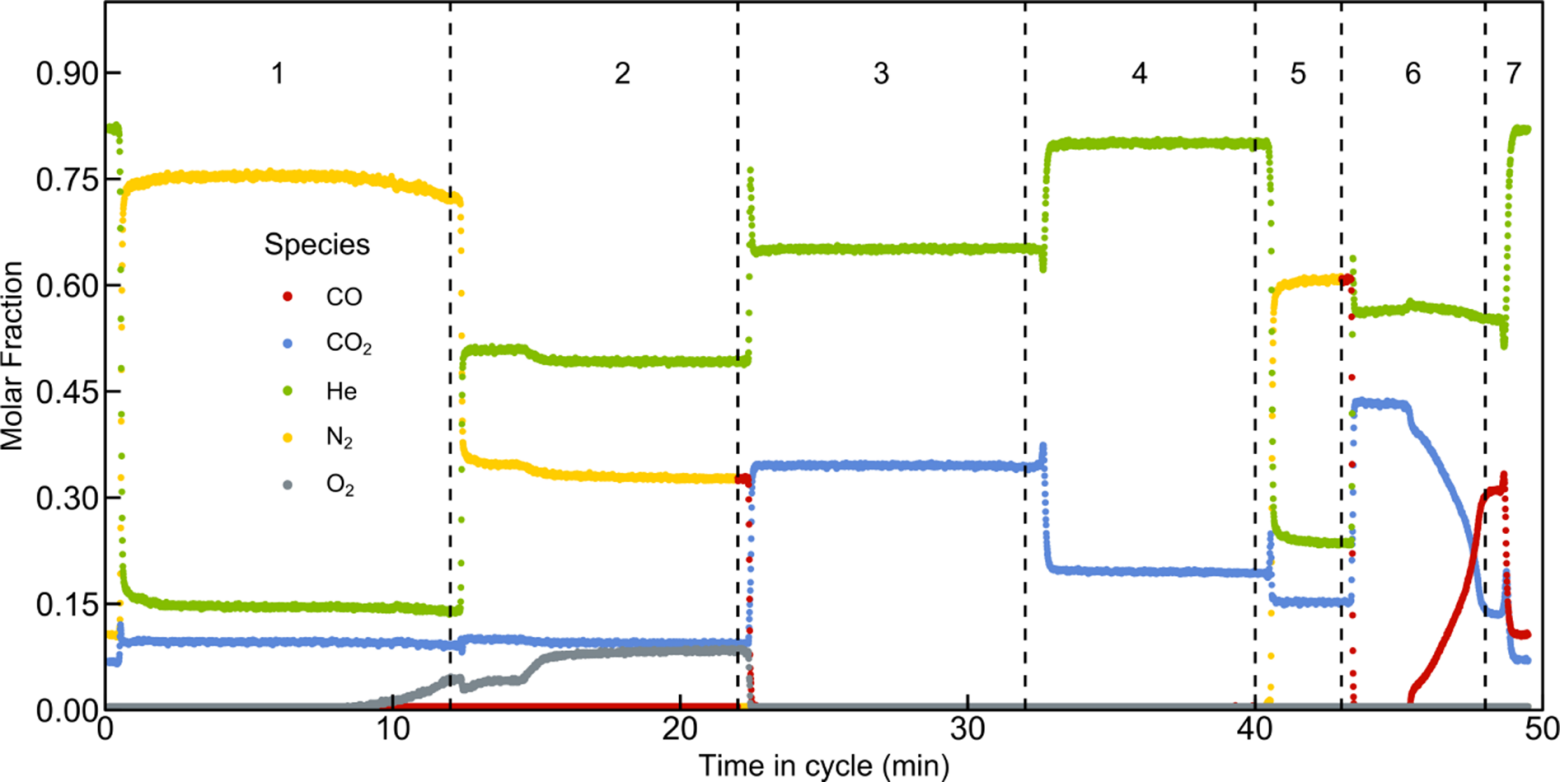
Product gas composition for a complete cycle with batched synthetic EAF off-gas (copper-based material, medium flow, 600 °C). Stage numbers correspond to the feed given in Table 1.

**Figure 6:**
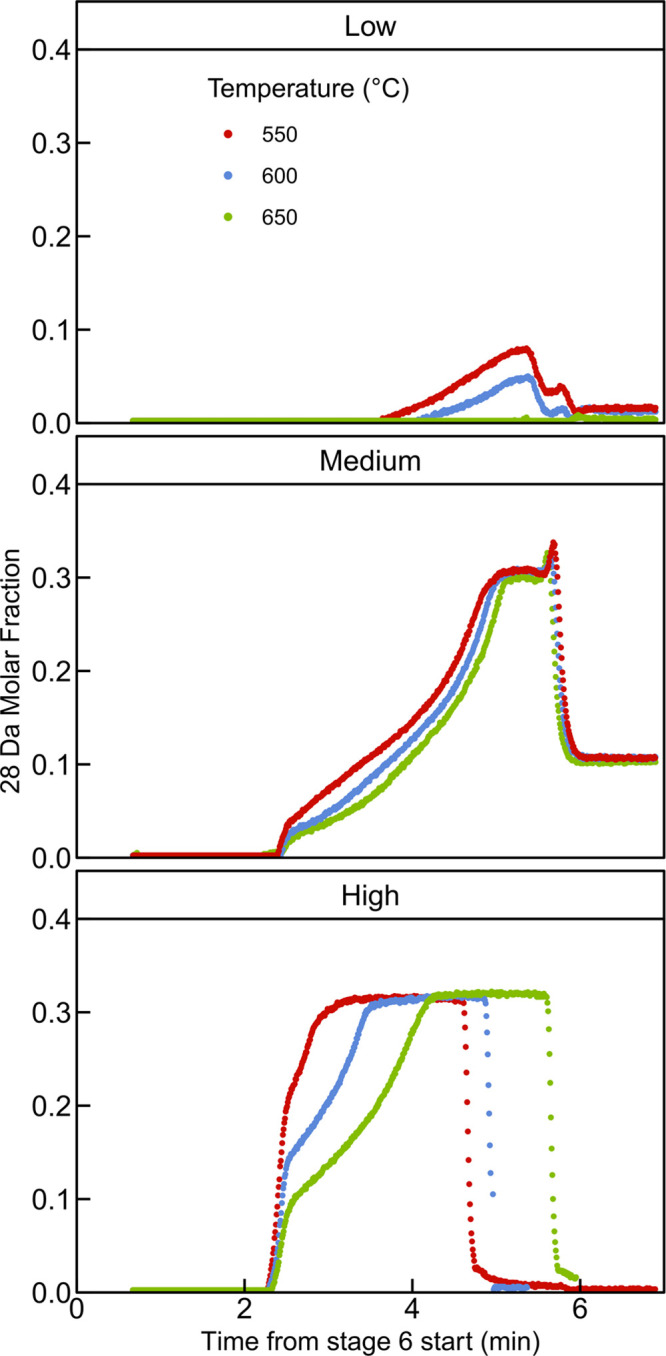
CO breakthrough for representative cycles of copper-based looping with low velocity batched gas flow, at low, medium, and high total flow rates and varying temperatures.

**Figure 7:**
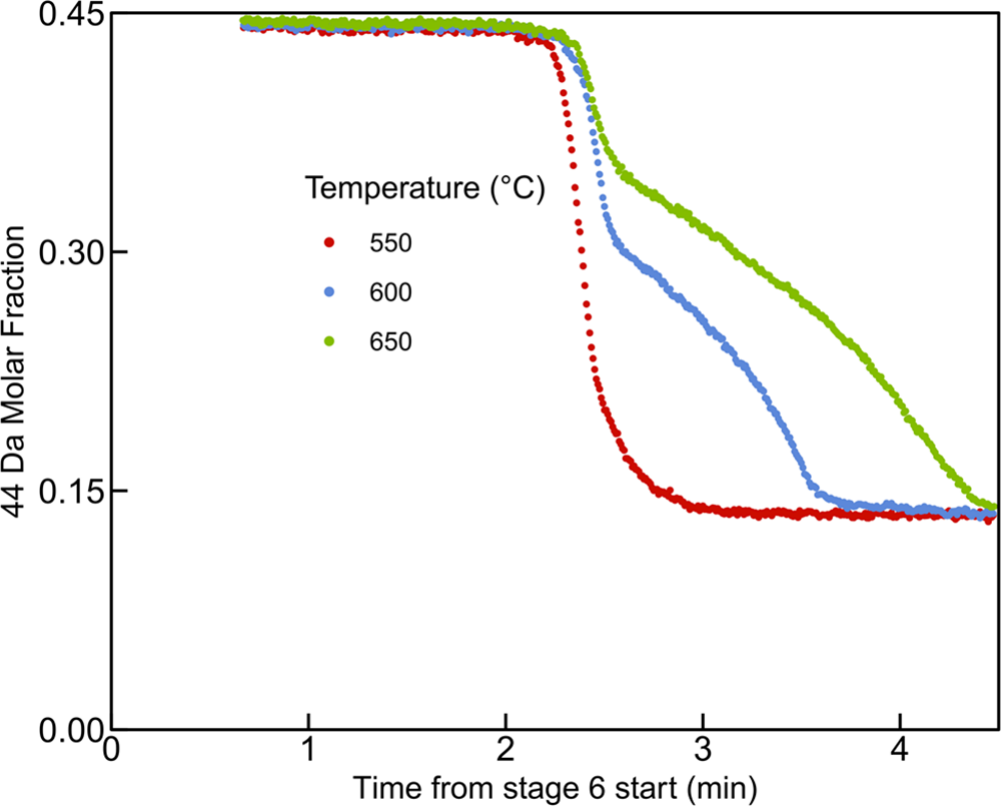
Reduction in CO_2_ in the product gases for the copper-based material under high flow conditions as the available bed oxygen is consumed.

**Figure 8:**
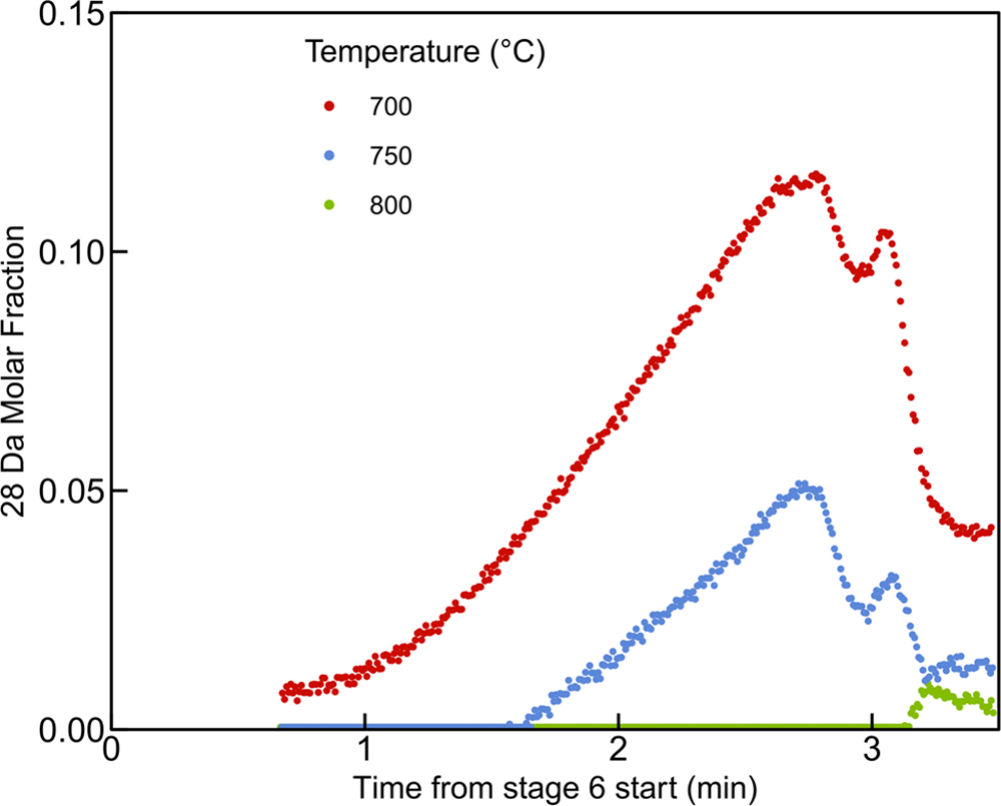
CO breakthrough for representative cycles of iron-based looping with high–high gas flow.

**Figure 9:**
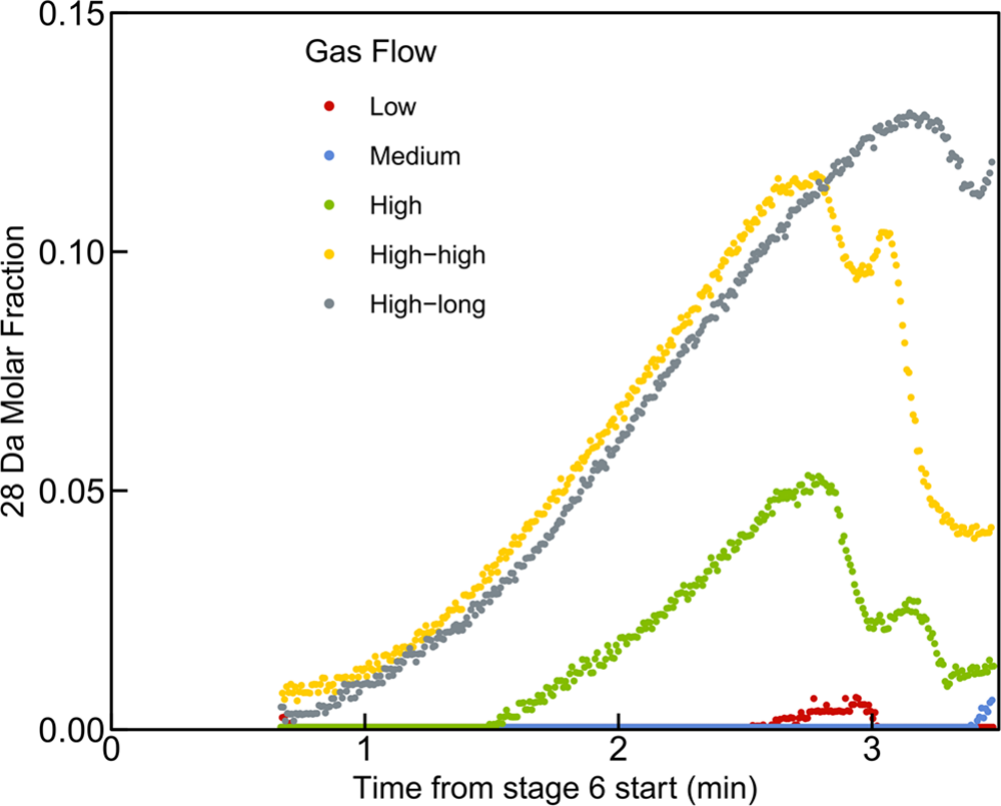
CO breakthrough for representative cycles of iron-based looping at 700 °C with varying batched gas flows.

The nominal bed conversion achieved with 95% confidence intervals (mean ± *t* × sd) before CO breakthrough to 5, 50, and 95% of the feed CO flow rate are given in Table 2 for the copper-based trials, and Table 3 for the iron-based trials. The number of cases with breakthrough observed are reported in Tables S2 and S3 for copper and iron, respectively. As previously noted, the values reported in these two tables do not consider data for the 28 Da MS signal from the first 40 s in stage 6 due to the presence of N_2_ carried from stage 5. This window was confirmed to not have apparent CO breakthrough in all trials, indicated by the baseline 28 Da MS signal. These results are based on five sample (2.5 s) rolling averages to reject noise. Bed conversions are based on the totalized flow of O_2_ and CO to the reactor. Breakthrough levels are reported on the estimated present-time outlet flow of CO against the present-time supply of CO.

**Table 2:** Nominal Solids Conversion at Breakthrough to 5, 50, and 95% of Feed CO Flow Rates with the Copper-Based OC

	low flow			medium flow			high flow		
temperature (°C)	5%	50%	95%	5%	50%	95%	5%	50%	95%
550	65.7 ± 2.3	71.0 ± 0.2	71.0 ± 0.2	68.5 ± 4.1	80.8 ± 4.2	87.2 ± 4.1	84.2 ± 12.4	85.4 ± 12.4	89.3 ± 15.3
600	69.3 ± 2.0	71.0 ± 0.0	NR	72.0 ± 5.5	86.0 ± 5.1	90.5 ± 5.3	85.4 ± 1.1	88.8 ± 1.1	95.7 ± 0.3
650	71.4 ± 0.7	NR^a^	NR	72.4 ± 1.4	87.5 ± 1.4	88.7 ± 0.3	82.7 ± 7.1	89.9 ± 4.7	97.4 ± 2.9

a NR = not observed/not reportable.

**Table 3:** Nominal Solids Conversion at Breakthrough to 5, 50, and 95% of Feed CO Flow Rates with the Iron-Based OC

	high flow			high–high flow		
temperature (°C)	5%	50%	95%	5%	50%	95%
700	51.1 ± 3.1	56.0 ± 1.1	56.6^a^	55.9 ± 3.9	69.8 ± 4.2^b^	68.8 ± 4.2^b^
750	56.3 ± 0.1	NR^c^	NR	59.7 ± 1.9	63.6 ± 1.1	65.4^a^
800	–^d^	–	–	64.5 ± 1.0	NR	NR

a Confidence interval not reportable (breakthrough only in a single trial).

b Breakthrough occurred between stages.

c NR = not observed/not reportable.

d – = trial not conducted.

Low levels of CO breakthrough were observed for the copper-based material at 550 °C and high flow rates late in stage 4 (first reduction) but were not observed for any other conditions with either material during the first reducing window. Breakthrough of O_2_ was not observed during stage 5 in any cycles, or early in stage 6 under any case.

### 3.2 Material Stability

Cyclic performance and repeatability for the two materials tested here are shown in Figure 10, and the material before use and once recovered is shown in Figure 3b,d for the copper and iron materials, respectively. For the copper material, cycles are shown at 650 °C under medium flow (material cycles 2–5, and 34–36). For the iron material, the cycles shown took place under high–high flow, at 700 °C (material cycles 21–24 and 44–45).

**Figure 10:**
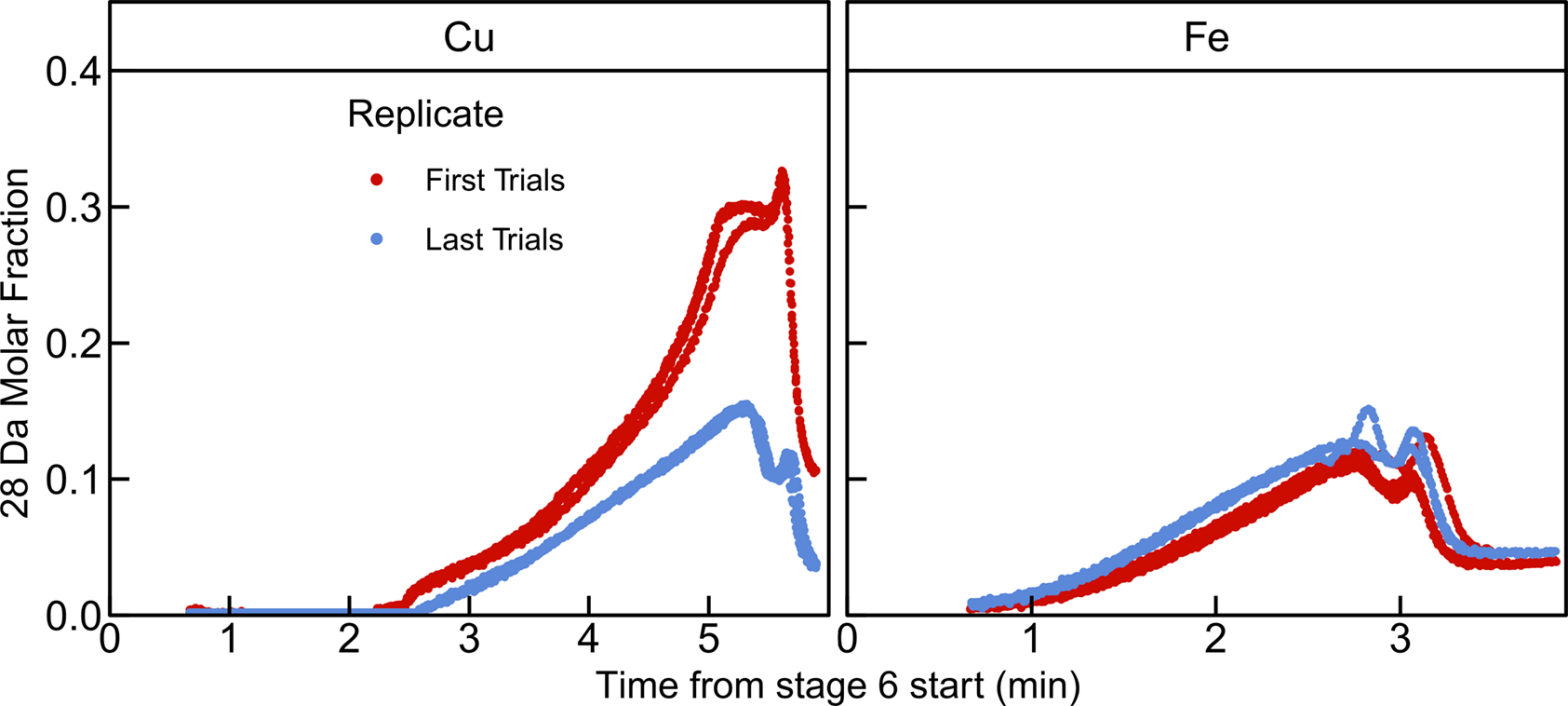
Extended cycle count CO breakthrough for the copper- and iron-based materials tested in this work.

### 3.3 Carbon Release

The release of carbon from the iron-based OC was observed as an increase in the estimated flow rate of CO_2_ during stages 1 and 2. Cases where this was observed are described in Table 4. Because carbon deposition is based on the previous cycle for reduction, data was filtered to exclude the first oxidation stage in each trial. No changes were noted in the flow of CO_2_ exiting the reactor with the copper-based material.

**Table 4:** Cases with Apparent Production of CO_2_ on Oxidation (Stages 1 and 2) with Cycling of the Iron-Based Material

temperature (°C)	low flow	medium flow	high flow	high–high flow	high–long flow
700	0%	0%	100%	100%	100%
750	–^a^	–	100%	100%	–
800	–	–	–	0%	–

a – = trial not conducted.

## 4 Discussion

### 4.1 Bed Temperature Effects

As expected, Tables 2 and 3 show that increases in the bed temperature resulted in increases in solids conversion before breakthrough. In the cases with copper, shown in Figure 6, the fraction as CO in the product gas exhibits a slower rate of change with increased temperatures during the breakthrough windows. This is most evident when comparing the 550 °C case with low flow to the 600 °C case, and the latter portion of the breakthrough period in the high flow cases at 600 and 650 °C. For iron, Figure 8 shows both the increased usable fraction of the bed and the extended window for breakthrough in response to higher temperatures, with the delayed onset for breakthrough with increasing temperatures, and the reduced rate of increase for the CO fraction in the product.

The increase in capacity and the extended window for CO breakthrough is readily explained by the improved reaction kinetics with increased temperatures. Studies on the reaction kinetics of OC materials such as copper and iron are plentiful in the literature. Derived reaction kinetics for a different copper-based material show a relative reaction rate increase of 25% when temperatures increase from 550 to 650 °C,^28^ which supports the results here, where 12–17% of the previously unused bed capacity was made available before 5% CO breakthrough was observed in the low and medium flow cases. The deviation between the improved reaction rates and increase in utilization may be due to three variations from the previous kinetic study; the kinetic work had no external mass transfer limitations, the OC previously used was approximately 1 order of magnitude smaller in diameter, and the active fraction was significantly changed from 10% Cu to be primarily Cu/CuO. Additionally, the materials may have had other changes in structure (e.g., specific surface area, porosity), further affecting reaction rates.

For a hematite (Fe_2_O_3_) sample, previous work has reported an activation energy for reduction by CO of approximately 20 kJ mol^–1^.^28^ For an increase in operating temperature from 700 to 750 °C, Arrhenius kinetics predict a 13% increase in reaction rate, while when increasing from 700 to 800 °C the reaction rate is 26% increased. In the high–high flow case with the iron, 8.6% of the residual bed capacity was used with the first 50 °C rise in temperature, while 20% of the remainder was used when moving to 800 °C. Again, the materials examined here were different from the previous work, but the improvement in bed utilization is clearly linked to reaction kinetics.

For the proposed application in an electric steelworks, preheating of gases, and therefore operating temperatures, is not a concern as gases are regularly produced at much higher temperatures than CLC requires. Rather, the gases produced by an EAF require cooling to reach temperatures safe for CLC. Gas quenching with liquid water spray is a low-cost possibility which could reduce and stabilize feed temperatures with minimal additional molar flow. Additionally, because the feed in the proposed application is dynamic and continuous, a fixed bed heat removal stage using a dedicated gas stream as previously proposed^29^ may not be possible and instead external heat removal is required. The diluting effect of a water quench would assist in minimizing bed temperature rises with unsteady cooling dependent on operating conditions. Control of this cooling flow would also improve the flexibility in bed operating temperatures, adjusting for long-term changes in reactivity.

Although iron materials typically have slower reaction kinetics than other common OCs,^1^ the ability to increase bed temperatures in designs with external heat removal to improve OC utilization shows potential for low-cost chemical looping installations. External heat removal limits vessel wall temperatures, giving a broader range of acceptable materials and reducing required wall thicknesses when following pressure vessel design guidelines.^30^ Additionally, EAF systems are driven by suction and can be mechanically designed for atmospheric or low vacuum operation. In contrast, other fixed bed chemical looping systems installations are typically designed for pressurized operation for kinetic optimization, requiring heavy vessels and higher capital expense. Alternatively, OC materials such as copper, and potentially manganese, give a lower-temperature option, albeit at higher cost, depending on operational needs and future materials development. The OCs used here offer potential to increase bed temperatures to increase effective capacity, if temperatures are not brought so high that sintering occurs. Higher-temperature materials such as nickel are also possible, given that the other considerations, including safety, bed utilization, reactor sizing, and cost are met. Overall, operating temperatures in the proposed application are not shown to be a hindrance or design limitation, but rather a flexible tool which can be adjusted for degradation of material or changing upstream operations in a facility with highly variable operations.

### 4.2 Flow Channeling Behavior

The results presented in Figures 6 and 7 indicate that flow channeling may have occurred, prompting early CO breakthrough and a reduction in CO_2_ concentration. In these cases, increased flow rates would have led to preferential flow through one or more partial-length channels (perhaps half the length of the full bed), leading to an accelerated reaction front in the channel(s) relative to the bed as a whole and subsequently being noted as early breakthrough of CO, and a sharp decrease in CO_2_ levels. In particular, the standard sigmoidal shape for breakthrough after an immediate rise indicates that a portion of the feed is passing through the bed unreacted, and mixing with gases which are inert or have been oxidized by the bed to CO_2_. This is particularly noticeable with higher gas velocities (creating a backpressure), and the copper material, which had a bed aspect ratio of approximately 2, and bed length to particle diameter ratio of approximately 30. Under pilot scale operations, such behavior would not be expected with reduced wall effects (as the ratio of particle size to vessel size decreases), increased operating time resulting in bed settling, and a longer reactor tube aspect ratio, with a minimum of 5–10.^31,32^ Additionally, reactor beds at pilot scales or larger will include entry and exit guard beds and flow distributors, reducing the likelihood of long channel formation. Even in the cases here using the iron-based material where the bed aspect ratio was approximately 3, this behavior was not observed.

These results may also be due to a combination of other reaction rate limits. The two materials investigated here have not been characterized for pore sizes, internal mass transfer rates, or surface reaction rates. The copper material, having been prepared on a support material, may have pores which have been or can become plugged. Alternatively, there may be a core–shell structure present which limits reaction rates beyond a critical local solids conversion. The iron material, having been prepared by extrusion from a blended powder, will have a different microstructure and uniform distribution of active oxides, likely limiting this behavior. Without further characterization it is not possible to determine if these effects are present.

### 4.3 Flow Rate Effects

Increases in the total flow rate to the reactor resulted in increased overall solids conversion before gas breakthrough was observed. In the cases presented here, the particle Reynolds number for the OCs indicates flow is within the laminar regime (Re_*d*_*p*__ on the order of 1–10), where external mass transfer limits are more prevalent and limiting to reaction rates than they would be with increased gas velocities. These limits are reduced by the increase in flow, with Tables 2 and 3 showing improvements in bed utilization for any temperature as flow rates increase. In full-scale systems, gas velocities of up to 0.85 m s^–1^ would be typical and have been tested in larger bench-scale systems,^8^ while the highest velocities here were 0.19 m s^–1^. Under the increased velocities expected of full-scale operations, with larger OC particle sizes to manage bed pressure drops, flow will be shifted to the transitional or turbulent regimes and external mass transfer limits will be reduced. However, if reaction kinetics or pore diffusion instead become limiting, earlier breakthrough of CO, H_2_, or hydrocarbons may be observed, especially as advective dispersion increases. This earlier breakthrough is comparable to the common fixed bed adsorption column, where increased fluid velocities result in lower bed utilization.^33^ To counter this, the length of the bed can be increased toward more typical design heuristics. In designs utilizing external heat removal, such as shell and tube designs like the configuration shown in Figure 4, individual tube aspect ratios will be suitably long.

The effects of bed conversion and flow rate are also seen in Figure 9. In these cases, the breakthrough of CO shows more sensitivity to the bed conversion than the gas velocity, with high flow under extended cycle timings mirroring the high–high flow case and even extending to higher levels of breakthrough. When paired with the mass transfer restrictions observed for cycles of the copper material, these results show that the breakthrough in dynamic cases results from a complex mixture of effects, and cannot be directly tied to bed conversion, temperature, or gas velocity.

### 4.4 Material Stability

Both the copper and iron materials showed excellent stability for the experiments presented here. Visually, Figure 3 shows some difference in both materials before and after use. A portion of the copper material exhibited changes in morphology, however Figure 10 shows that the material was activated by early cycles, with improved reduction reaction kinetics in later cycles as indicated by a lower level of CO breakthrough. There is also what appears to be migration of copper, with some support material visible in Figure 3b, as indicated by a lighter color. Under the limited conditions tested here, this material appears to be a promising candidate for scale-up.

The iron-based material shows minor morphological changes after use, with some cracking in the structure visible in Figure 3d, especially in low aspect ratio (short) particles. In Figure 10 there is a small decrease in evident reduction reaction rate, with later cycles having a slightly elevated breakthrough curve (*p* < 0.05), with an average CO level 1.3 ± 0.07% higher in the product gas for later cycles than for the earlier cycles. These two results indicate a small decrease in the quality of the iron-based material, which may require a higher replacement rate in full-scale operations. This replacement rate would be offset by the low material cost, being derived from steelmaking scrap and installed in a steelmaking facility.

In evaluation of the OCs used here, it is also important to consider that the material microstructure could not be characterized, and further questions remain. There may have been pore blockage or phase migration which were hindering performance, which could be examined for other materials by electron microscopy. Additionally, full-scale operations will most likely have much higher gas velocities—noted to be a source of attrition in fluidized bed looping and tested for in jet cups^34^—and much higher cycle counts, expected to exceed 5000 cycles year^–1^ for an industrial unit. Despite this, degradation in fluidized bed looping has been most notable on startup,^35,36^ which indicates that the materials tested here may be viable candidates for long-term, industrial scale operations given the relatively minor degradation seen here. Further TGA and electron microscopy of any materials for this application are recommended to examine their stability, with bench-scale testing of gas flow attrition resistance.

### 4.5 Coke Formation

#### 4.5.1 Presence of Reaction

Carbon deposition as coke was observed with the iron-based material, despite the lower overall bed conversion than for the copper-based material. Previously, Cho et al.^37^ noted that a nickel-based material demonstrated coke deposition, but for an iron-based material no coke formation should be expected. For combustion of CO over OCs, they further state that less coke can be expected to be formed than for combustion of CH_4_. The deposition of coke comes from the Boudouard reaction, reaction 1:^38^

$$2\mathrm{CO}\overset{\mathrm{catalyst}}{⇌}{\mathrm{CO}}_{2}+C$$ 1

Reaction 2, for the removal of coke during the oxidation stage of the cycle, is also used to determine if coke is formed through the difference in CO_2_ flow in and out of the reactor:

$$C+O_{2}\to CO_{2}$$ 2

The results presented in Table 4 support that reactions 1 and 2 are taking place, as the forward component of reaction 1 (producing coke) is favored by lower temperatures, and was demonstrated at 700 and 750 °C, but not 800 °C. It could not be determined if the reverse reaction of reaction 1 occurred, due to the presence of N_2_ in the product gas, and/or the subsequent oxidation of any CO produced further along the bed.

Reaction 1 is only occurring with the iron-based material as it is a catalytic reaction. Typical catalysts described in literature include nickel and its oxides, as well as molybdenum oxides,^39^ iron and its oxides,^40^ and cobalt oxides,^41^ however coking is a well-reported phenomena with catalysts more generally.^42^ Notably, the signs indicative of reaction 2 late in stage 1 (oxidation) of the cycle show that reaction 1 is taking place in the bed, and not with the iron or nickel present in the reaction vessel, which would result in an early rise in CO_2_ outflow. Coke formation also only occurred with higher extents of bed reduction (high flow, high–high flow, and high flow with extended cycle time cases) when there was a longer residence time for CO over catalytic materials in the bed, prior to oxidation by Fe_2_O_3_. This may also be due to lower oxidation states of iron (e.g., FeO, Fe_3_O_4_) being better catalysts for reaction 1 than Fe_2_O_3_. In future studies, creating layers of packing separated by quartz wool or another inert material would assist in recovery of the OC based on its oxidation state. This would support determination of the mechanism of coke deposition, and the bed conversion required for coke to appear. Alternatively, targeted TGA studies may support investigation of coke formation.

On oxidation, the late appearance of additional CO_2_ shows that the OC was preferentially oxidized over coke deposits, indicating poor reaction kinetics for reaction 2. The recovery of coke seen in Figure 3d further supports this, as the OC had been oxidized for an extended window, which under ideal operations would remove any coke deposits.

#### 4.5.2 Impact on Operations

The formation of coke by catalysis on iron provides support for use of either copper or manganese as an active species in OCs for use in the proposed EAF application. The copper-based OC did not demonstrate coking, and manganese is not noted in the literature to be prone to coking, although this is perhaps due to a lower usage frequency, or under conditions where reaction 1 is avoided. By reducing the formation of coke, CO_2_ separation is improved, and total emissions can be reduced when coupled with CCUS by preventing slip to the nonconcentrated air stream in bed regeneration. Additionally, prevention of coke formation in this application will reduce the need for maintenance to keep the bed in its target kinetic region. Regeneration with steam—as shown in Figure 4—or concentrated O_2_ is possible, however this requires additional capital and operational expenses, as well as process downtime. The use of a water quench for temperature management, as previously discussed, would also assist in removing any coke deposits via reaction 3. Finally, there is the possibility of OC replacement, however this is an expensive and maintenance-heavy operation compared to fluidized bed CLC where supplemental OC can be added without shutdown.

$$C+H_{2}O\to \mathrm{CO}+H_{2}$$ 3

### 4.6 Dynamic Fixed Bed Chemical Looping

Most studies to-date for fixed bed chemical looping at the bench-scale have focused on gas-switching, using two or three stages, in some cases to produce heat but more commonly for reforming reactions. This work presents the first steps in moving to cases where well-defined stages do not exist, using seven shorter steps instead of longer cycles with alternating gases present. Variations in flow, and the use of a short midcycle oxidation (stage 5) which is representative of perhaps a trip or process bump, have demonstrated that fixed bed chemical looping is robust, and can be used as a means of converting dynamic waste streams which would otherwise need multiple treatments or would be flared, into a product which is below CCUS pipeline transport limits of 0.1% CO, 4% H_2_, and approximately 5% hydrocarbons.^43,44^ Although H_2_ and hydrocarbons have not been investigated here, the increased tolerance for their presence in CO_2_ pipelines, and considering their presence as an average concentration across a cycle, show potential for fixed bed CLC as a treatment method of these contaminants. Depending on the source of the gas stream, other OCs may be more suitable, such as Ni/NiO in conversion of CH_4_.^1^ Additionally, as shown in this work, reactor temperatures in robust designs can be increased to improve gas conversion.

In development of pilot or industrial fixed bed chemical looping for dynamic gas conversion, systems do not necessarily need to be designed for 100% up-time, if flares or scrubbing systems are already in place and are kept in a ready state. This can allow for heat recovery from waste gases with concurrent emissions reduction in existing installations, with off-spec CLC products being treated by conventional means and vented. However, as the results here show, gas conversion is generally very high in this operating mode, with CO below detection limits despite its presence up to one-third of the reactor feed. If reactors are sized according to expected gas flows alternative treatment may not be necessary outside of special circumstances (e.g., OC activation, cold reactor startup, coke removal).

Other processes which have dynamic gas products are abundant, although they are not always waste gases. This is especially notable in batch processes, with startup and shutdown windows. Previous experimental investigations of gasification have shown that the product gas composition is variable, like the EAF case presented here, but also subject to the variability in feedstock.^45^ Other raw materials refining processes, such as aluminum smelting,^46^ cement production,^47^ and pyrolysis^48^ produce gases containing a dynamic flow of CO, H_2_, or hydrocarbons, depending on process conditions and specific feedstock. Present practice for these sectors is the release of product gases, either pure or diluted by air, and/or flaring in an attempt to limit hydrocarbon release while recovering some heat, but limiting potential for CCUS. Heat recovery from the combustion of the gases in these batch processes by chemical looping is possible, and investigation of these dynamic gases as feed for fixed bed chemical looping warrants investigation. However, other contaminants present in these gas streams, such as sulphurous species or halocarbons, may make capture difficult to economically justify to investors. These species may also be damaging to OCs in long-term operations.

Outside of batch processes, continuous processes also experience dynamic operations and their associated challenges, especially when using natural feedstocks. One case where a small fixed bed reactor may be viable as a gas cleaning technology is the oxy-fuel combustion of coal. Presently, oxy-fuel combustion is operated with excess O_2_ for complete combustion of CO,^49^ however this is an inefficiency in the process which requires deoxidation later.^50^ A fixed bed CLC system could allow oxy-fuel combustors to operate at expected stoichiometric flows, with excess O_2_, CO, or H_2_ being reacted in the CLC unit, rather than relying on active control. If the molar composition for any of these three species is kept below 1% typical, small reactors with limited OC inventories would be all that is required. If long-term (on the order of minutes) flow containing O_2_, CO, H_2_, or others is observed, such a system provides enough time for slower-acting control to adjust feed ratios. Outside of cleaning oxy-fuel combustor products, fixed bed CLC could find use in fluidized bed CLC installations, which have been shown to have low levels of incomplete combustion products exiting their fuel reactors.^4^ Catalytic or in-line combustion have been proposed for gas cleaning,^51,52^ which requires active measurement and control. The hot, vitiated air product could be used as a means of slowly reoxidizing any beds.

In another application, fixed bed chemical looping could be installed as a treatment step for flue gas polishing in existing coal, oil, or natural gas power plants, where fugitive CH_4_ emissions are noted to currently be—and are expected to remain—a major contributor to climate change.^53^ Fixed bed CLC, as previously noted, offers an alternative to catalytic combustion which can maintain low levels of oxidizable species such as CH_4_, reducing the global warming potential of existing emitters while adding minimal additional process equipment. Cu_2_O, one of the OC materials investigated here, has also been shown to be capable of reducing NO_*x*_,^54^ offering further potential for flue gas cleaning in existing combustors without the use of expensive catalysts, or active management.

In cases where the bed is not regenerated by an O_2_-containing stage, a return to basic gas-switching step with ambient air supplied as an oxidizer is possible. In this case, the residual heat contained in the bed can bring the makeup air to reaction temperatures, completing the loop. Depending on the nature of the process, this step may or may not require additional reactors; in batch processes with expected downtime a single reactor may be suitable, while when used for continuous processes multiple reactors with gas switching may be needed. In these cases, it is not imperative that reactors operate with carefully timed gas switching, eliminating the engineering challenges associated with fast switching of gases, a current roadblock to fixed bed chemical looping.

In single reactor systems upstream gas switching and its challenges are eliminated, however there will still be a need for downstream gas switching, to either vent O_2_-containing gases (heat removal and oxidation stages), or to capture CO_2_-containing gases (reduction). Unlike upstream gas switching, in the configuration shown in Figure 4 the gases are available to a CO_2_ purification system (e.g., adsorption, membrane separation, amine systems) at a lower temperature, while not containing flammable gases. Under these conditions valves do not require specialty materials or high-temperature hazardous location actuators and can readily be sourced. To manage the variability from the process and improve the performance of separations, a flow buffer system (large, low-pressure tank) downstream of the chemical looping reactor can assist in dampening changes in composition and flow, while helping to remove any condensed H_2_O depending on the upstream process.

It should also be noted that the proposed system may not be capable of total capture of CO_2_ emissions from a dynamic process. There is the possibility of dilute CO_2_ in the oxidizing feed (see stages 1 and 2 of Table 1), and CO_2_ losses from the delay time in measuring O_2_ and CO_2_ levels before actuating downstream valves and directing CO_2_ to purification. However, the proposed design can capture most of the CO_2_ from a dynamic CO/H_2_/hydrocarbon gas stream and is a marked improvement in producing a CO_2_-rich gas over in-line combustion with dilution air, as is employed by EAFs at present. This minimizes the total flow of gases to CO_2_ purification and CCUS, while maximizing the CO_2_ concentration, to reduce the capital and operating expenses of the process.

## 5 Conclusions

A process configuration which can integrate fixed bed chemical looping for the conversion of dynamic gas streams found in industry has been proposed. The effects of bed temperature on OC utilization have been highlighted, with nominal solids conversions exceeding 60% being shown to be viable with greater than 95% conversion of synthetic EAF off-gas. Higher utilizations are possible by increasing bed temperatures in a reactor with external heat removal. Increased gas velocities resulted in increased effective reactor capacities, however these results have yet to be demonstrated at larger scales, where advective dispersion will lead to earlier breakthrough. These results also indicate potential flow channeling for a portion of the bed length, magnified by increases in flow rates, but likely to not be a concern as reactor scales are increased.

The copper-based material tested was shown to be stable for the conditions used, with improved reduction reaction kinetics as cycle counts increased; the iron-based material showed some deactivation, although it is unclear how this will affect long-term operations. Notably, the formation of coke on iron-based OCs was observed and is proposed to be occurring as a catalytic reaction which may also occur with other OC materials. Coke formation is proposed as one possible reason for apparent deactivation of the iron-based OC. Coke formation is also considered for its reduction in overall carbon isolation, which is a core function of chemical looping processes. For this reason, further investigations of the material are proposed.

The experiments presented here thus serve as a first step in demonstrating that fixed bed chemical looping reactors can operate in more configurations than a two-step or three-step gas switching process, processing the dynamic off-gas produced by continuous and batch industrial processes as a source of fuel, while producing a product with CO concentrations below 2% of CO_2_ transport pipeline limits, and with minimal required active control or management. A downstream gas switching step may be required, however it has been noted that this can be completed cold, and may not need to be a fast or carefully timed switch. H_2_ and hydrocarbon oxidation were not examined, but with appropriate selection of OC and operating conditions, and higher acceptable levels in CO_2_ transport, treatment of dynamic streams to pipeline specifications can be reasonably expected. The synthetic batched EAF off-gas used here is shown to be treatable under tightly controlled conditions (low gas velocities, step-changes in flow, minimal bed temperature rise), but limitations to the study are considered. Overall, these results show further investigation is warranted for chemical looping with dynamic feed, and for chemical looping as a means of reducing the environmental footprint of the EAF.

## Supplementary Material



## Notes

Expanded information on experimental trials and cases showing gas breakthrough (PDF)

## ⊥

Department of Chemical Engineering, University of Bath, Bath BA2 7AY, United Kingdom

## Abbreviations

CCUS: carbon capture, utilization, and storage
CLC: chemical looping combustion
EAF: electric arc furnace
MS: mass spectrometry
OC: oxygen carrier
SCCM: standard cubic centimeter per minute
TGA: thermogravimetric analysis
