# Supplementary material for: Fixed Bed Chemical Looping beyond Gas Switching: Application to Dynamic Industrial Waste Gas Conversion
Source: Ind Eng Chem Res. 2026 May 27;65(22):11618–30. doi: 10.1021/acs.iecr.6c00301 (PMC13262049; doi:10.1021/acs.iecr.6c00301)
Supplement: Supplementary file 1 [file ie6c00301_si_001.pdf]

Fixed Bed Chemical Looping Beyond Gas Switching: Application to Dynamic Industrial Waste Gas Conversion - Supplemental Material

Andrew J. Furlong,[a,b,c*] Nicole K. Bond,[a] Jan B. Haelssig,[d] Christopher de Leeuwe,[c†] Adam Zaidi,[c] Michael J. Pegg,[b] Vincenzo Spallina,[c] Robin W. Hughes[a]

[a] Natural Resources Canada, CanmetENERGY-Ottawa, 1 Haanel Drive, Ottawa, ON, K1A 1M1, Canada

[b] Department of Process Engineering and Applied Science, Dalhousie University, PO Box 15000, Halifax, NS, B3H 4R2, Canada

[c] Department of Chemical Engineering, University of Manchester, Manchester, M13 9PL, United Kingdom

[d] Department of Chemical and Biological Engineering, University of Ottawa, Ottawa, ON, K1N 6N5, Canada

[*] Email: afurlong@dal.ca

[†] Present affiliation: Department of Chemical Engineering, University of Bath, Bath, BA2 7AY, United Kingdom

Table S1. Order of experimental trials.

Trial number	Oxygen carrier	Flow level	Temperature (°C)	Cycle count	Cumulative cycles
1	Copper	Medium	650	5	5
2	Copper	Medium	600	5	10
3	Copper	Low	600	3	13
4	Copper	Medium	550	5	18
5	Copper	Low	550	3	21
6	Copper	High	550	3	24
7	Copper	High	600	3	27
8	Copper	High	650	3	30
9	Copper	Low	650	3	33
10	Copper	Medium	650	3	36
11	Iron	Low	700	10	10
12	Iron	Medium	700	5	15
13	Iron	High	700	5	20
14	Iron	High-high	700	4	24
15	Iron	High-high	800	5	29
16	Iron	High-high	750	4	33
17	Iron	High	750	5	38
18	Iron	High-long	700	4	42
19	Iron	High-high	700	3	45

Table S2. Fraction and number of cases showing breakthrough of CO to 5 %, 50 %, and 95 % of feed volumetric flows using the copper-based material.

Temperature (°C)	Low flow			Medium flow			High flow		
	5 %	50 %	95 %	5 %	50 %	95 %	5 %	50 %	95 %
550	100% (3)	100% (3)	100% (3)	100% (5)	100% (5)	100% (5)	100% (3)	100% (3)	100% (3)
600	100% (3)	67% (2)	0% (0)	100% (5)	100% (5)	100% (5)	100% (3)	100% (3)	100% (3)
650	100% (3)	0% (0)	0% (0)	100% (6)	100% (6)	100% (6)	100% (3)	100% (3)	100% (3)

Table S3. Fraction and number of cases showing breakthrough of CO to 5 %, 50 %, and 95 % of feed volumetric flows using the iron-based material.

Temperature (°C)	High flow			High-high flow		
	5 %	50 %	95 %	5 %	50 %	95 %
700	100% (5)	100% (5)	20% (1)	100% (6)	100% (6)	100% (6)
750	100% (3)	0% (0)	0% (0)	100% (4)	100% (4)	25% (1)
800	-	-	-	100% (5)	80% (4)	0% (0)

- = Trial not conducted.
